# Hot Deformation Behavior and Processing Maps of 26CrMo7S Steel Used in Oil Exploration

**DOI:** 10.3390/ma16217056

**Published:** 2023-11-06

**Authors:** Hemiao Jiang, Hongying Li, Dianyuan Huang, Yinghui Zhao, Jiwen Liu, Qing Gao, Hang He, Ximao Liu

**Affiliations:** 1 School of Materials Science and Engineering, Central South University, Changsha 410083, China; hemiaojiang028@hotmail.com; 2 Hengyang Valin Steel Pipe Co., Ltd., Hengyang 421001, China; hdianyuan@163.com (D.H.); 18874125065@163.com (Y.Z.); hehang2003@163.com (H.H.); 3 Xiangtan Iron & Steel Co., Ltd. of Hunan Valin, Xiangtan 411101, China; ljw-0707@163.com (J.L.); 212671@mail.hnxg.com.cn (Q.G.); 206291@mail.hnxg.com.cn (X.L.)

**Keywords:** hot deformation, constitutive equation, dynamic recovery, dynamic recrystallization, hot processing map

## Abstract

The hot deformation behavior and flow stress characteristics of experimental 26CrMo7S steel were analyzed using a thermal simulator under a range of conditions, including a strain rate range of 0.01~10 s^−1^, a temperature range of 850~1250 °C, and a maximum deformation amount of 70%. The Arrhenius constitutive model was built for the corresponding conditions, and the model’s accuracy was verified through error analysis. Additionally, hot processing maps were constructed to analyze the processing zone of the steel under different hot deformation conditions. Finally, the microstructure of the processing zones was observed and verified using the electron backscattered diffraction (EBSD). The results indicate that the interaction of work hardening and dynamic softening influences the hot deformation behavior of 26CrMo7S steel. The Arrhenius constitutive equation with a value of the correlation coefficient (*r* = 0.99523) accurately predicts the flow behavior of 26CrMo7S steel under different strains. The optimal processing zone obtained with the hot processing maps is within a deformation range of 1010~1190 °C and a strain rate range of 0.01~10^−1.5^ s^−1^, and the obtained microstructure is in good agreement with the analysis results.

## 1. Introduction

26CrMo7S steel is a corrosion-resistant material widely used in oil exploration. With the decrease in traditional oil resources, unconventional oil fields like shale and tight oil are being explored. Deeper oil wells mean increased temperature and pressure, leading to a more adverse operating environment for oil well pipes, resulting in the need for steel with higher safety and reliability standards [1,2,3]. The appropriate hot working parameters can benefit steel by achieving a uniform and stable microstructure and good strength, greatly influencing the quality of final products in industrial production [4,5,6,7,8,9]. To simulate the hot deformation process, a thermal simulation experiment can be conducted. This experiment can create a constitutive equation that correlates stress, strain, strain rate, and deformation temperature. This equation is then used to predict the flow stress and the changes in the microstructure during hot deformation. By establishing a constitutive model, it becomes easier to set reasonable hot deformation parameters and reduce the need for extensive experimental work [10]. Therefore, it is essential to develop a reasonable constitutive model to describe the deformation behavior and predict the flow stress [11,12].

The study of hot processing maps helps control microstructure and ensure optimal performance by avoiding generating defects caused by inappropriate hot deformation parameters. In recent years, experts have studied different alloys to create models and maps for hot processing. Zhou et al. [13] tested the Inconel 718 superalloy during hot deformation compression using a Gleeble-3500 thermal simulation test system. Using stress–strain data, they developed the strain-compensated Arrhenius constitutive equation, which can accurately describe the hot deformation behavior of the investigated superalloy. Shao et al. [14] examined how hot isostatic pressing (HIP) impacted 9Cr oxide dispersion strengthened steel. They investigated a range of deformation temperatures, from 950 to 1100 °C, and strain rates, from 0.001 to 1 s^−1^. The research found that fully recrystallized structures corresponded to a high-energy dissipation zone. Zhang et al. [15] studied the hot deformation behavior of S460ML steel and developed a mathematical model to predict the critical strain of dynamic recovery (DRV). This model served as a guide for actual engineering analysis. Zhang and colleagues [16] performed compression tests on the Cu-Cr-Zr-Y alloy under isothermal conditions. The tests were conducted within a temperature range of 650~850 °C and a strain rate range of 0.001~10 s^−1^, using the Gleeble-1500D thermal simulation test machine. they were able to develop stress–strain curves, a complex flow stress constitutive equation, and hot processing maps. Furthermore, they determined the optimal hot deformation parameters within the temperature range of 800~850 °C and strain rate range of 0.01~1 s^−1^. These studies showed that the establishment of a constitutive equation and the use of hot processing maps can accurately predict deformation mechanisms and prevent processing instability of material during hot deformation. In addition, the constitutive and hot processing maps are beneficial to identify the appropriate hot deformation parameters of material and provide valuable guidance for safe process and gaining the suitable performance of material during the hot deformation. Although numerous studies concern the constitutive models of various alloys merged with hot processing maps, there is a lack of systematic research on 26CrMo7S steel, and the microstructure and final properties of 26CrMo7S steel are affected by hot deformation conditions. Therefore, it is necessary to study the hot deformation parameters of 26CrMo7S steel and derive a corresponding constitutive model and hot processing maps, facilitating the optimization of hot deformation parameters for this particular steel.

In this paper, a high-temperature simulation compression test was conducted on the 26CrMo7S steel sample. The true stress–strain curve was plotted from the experimental data, and then the Arrhenius constitutive equation was modeled. According to the curve and the model, the distribution of stress, strain, and temperature fields, as well as the relevant flow properties, were investigated under different hot deformation situations. The relationship between flow stress and the deformation process was analyzed quantitatively, and hot processing maps were created to examine the cooperative deformation and microstructural characteristics of 26CrMo7S steel. These results provide a mathematical model for predicting flow stress under different hot deformation conditions and a theoretical basis for obtaining suitable properties of experimental steel through the building of hot processing maps.

## 2. Materials and Experimental Procedures

The experimental material is 26CrMo7S steel, and its chemical composition is shown in Table 1.

**Figure 1:**
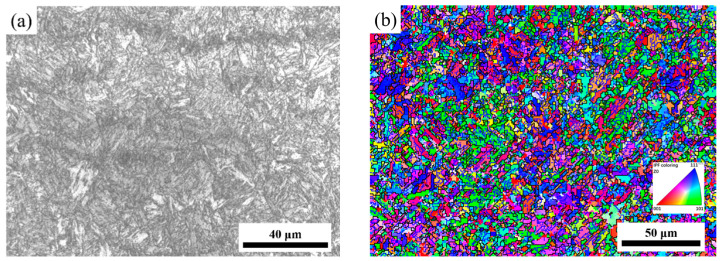
The images of microstructure of experimental steel: (**a**) OM; (**b**) IPF map.

Figure 1a,b show the images of the microstructure of experimental steel before hot deformation tested with optical microscope (OM) and electron backscattered diffraction (EBSD), respectively. The metallographic organization (Figure 1a) presents the homogeneous tempered organization, and the inverse pole figure (IPF) (Figure 1b) shows the fine grains of uniform distribution in the experimental steel.

**Figure 2:**
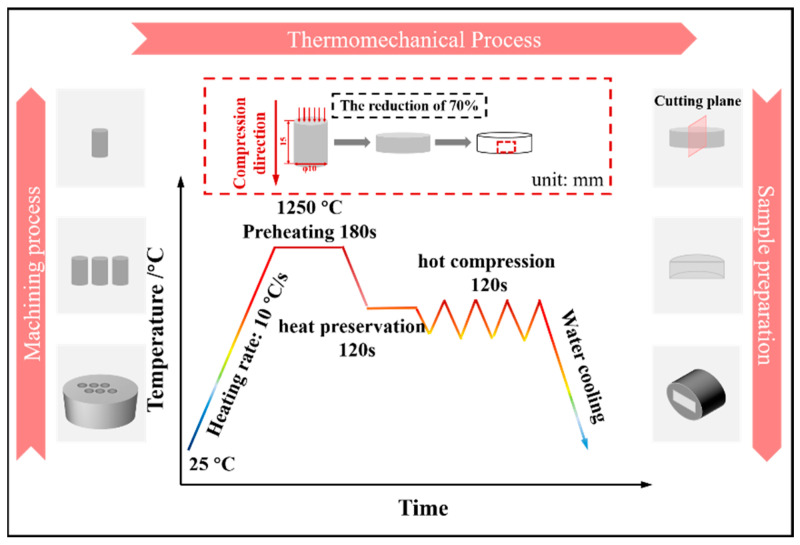
Hot deformation process of 26CrMo7S steel.

The experimental procedure and the hot compression test carried out on a Gleeble-3800 thermal simulation testing machine are presented following the schematic map in Figure 2. The experimental specimens with a diameter of 10 mm and a height of 15 mm were cut from the 26CrMo7S steel [17,18]. As depicted in the machining process/left side of Figure 2. To ensure uniform deformation during the hot compression test and minimize friction between the specimen and the indenter, high temperature lubricant in the form of mica flakes was applied on both ends of the specimen. The curve within Figure 2 show the thermomechanical process. Initially, the specimens were preheated to 1250 °C at a rate of 10 °C/s and held at this temperature for 180 s to complete austenitization. Subsequently, they were cooled to the deformation temperature at the same rate and held for 120 s, following which isothermal compression began. After hot compression, the specimens were cooled in water to room temperature. In order to analyze the microstructure, all specimens were cut along the axial cutting plane (as depicted in the sample preparation section of Figure 2) and then mounted, ground, and polished.

## 3. Results and Discussion

### 3.1. Flow Stress–Strain Behavior

**Figure 3:**
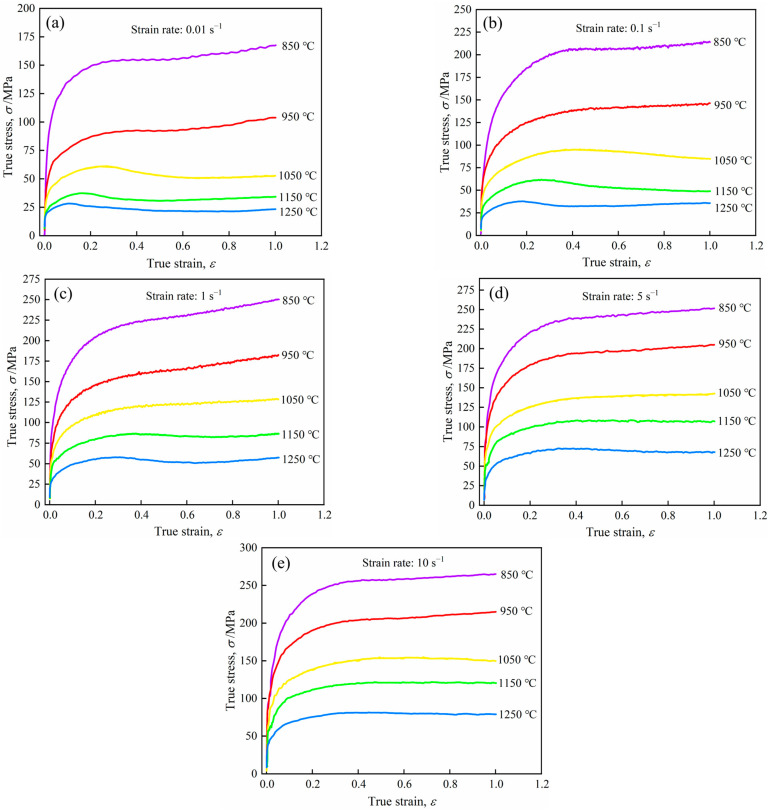
Stress–strain curves of 26CrMo7S steel at different strains with a strain rate of (**a**) 0.01 s^−1^; (**b**) 0.1 s^−1^; (**c**) 1 s^−1^; (**d**) 5 s^−1^; (**e**) 10 s^−1^.

The true stress–strain curves of 26CrMo7S steel are depicted in Figure 3. These curves illustrate the effect of deformation parameters on flow stress and reflect the dynamic behavior of steel during hot deformation. It can be seen that the stress–strain curve of 26CrMo7S steel shows a sharp rise at first [19,20]. The deformation temperature is relatively low at this stage, and the softening effect is insufficient. As a result, the work hardening caused by dislocation generation and tangle cannot be entirely offset, causing the curve to rise. It then gradually stabilized until the end of the test, which implied a balance between work hardening and dynamic softening [21,22,23]. The flow stress values became larger as the strain rate increased from 0.01 s^−1^ to 10 s^−1^. But with the deformation temperature increased from 850 °C to 1250 °C, the flow stress value gradually decreased.

When the strain rate was 0.01 s^−1^ and 0.1 s^−1^, the flow stress curve exhibited a dynamic recovery type at deformation temperatures of 850 °C and 950 °C. When the deformation temperature was 1050 °C or above, the flow stress curve exhibited dynamic recrystallization, the same result as Chen et al. [24], and they and Imbert et al. [25] suggested that DRX behaviors occur easily at a high deformation temperature because it lowers the driving force, but raises the occurrence frequency and the grain boundary mobility. Whereby the softening effect was stronger than the hardening effect, leading to stress reduction. 

When the strain was 1 s^−1^, at a deformation temperature of 850~1050 °C, the flow stress curve increased with increasing strain, displaying dynamic recovery [24,26], while the flow stress curve with a deformation temperature of 1150 and 1250 °C demonstrated dynamic recrystallization. When the strain rate was 5 and 10 s^−1^, the flow stress curve exhibited a dynamic recovery type at deformation temperatures ranging from 850 °C to 1250 °C. As the strain increased, the flow stress peaked and demonstrated a tendency towards flattening or continuous rise.

### 3.2. Constitutive Equation

The constitutive equation can determine the relationship among the deformation parameters, such as flow stress, temperature, and strain rate. The strain rate was controlled by the hot activation process, which occurred during hot deformation. Flow stress is essential data for high-temperature flow characteristics, while deformation temperature and strain rate greatly influence it.

#### 3.2.1. Stress Parameters

Based on the true stress–strain curve, the relationship between flow stress and deformation parameters was often expressed using the classical Arrhenius constitutive model.

The Arrhenius constitutive model proposed by Sellars and Tegart is widely acknowledged for its ability to demonstrate the correlation among flow stress, temperature, and strain rate [27]. The Arrhenius model is used to characterize the relationship between flow stress and deformation parameters, as shown in Equation (1) [28,29,30].

(1) $$Z=\varepsilon ˙\exp\left(\frac{Q}{RT}\right)$$

The Arrhenius constitutive equation can be expressed in three equations following the different stress levels. The constitutive equation of 26CrMo7S steel is based on the following three forms, respectively [31].

When ασ<0.8,

(2) $$\varepsilon ˙=A_{1}\sigma^{n_{1}}\exp(-\frac{Q}{RT})$$

When ασ>1.2,

(3) $${\varepsilon ˙=A}_{2}exp(\beta \sigma )\exp(-\frac{Q}{RT})$$

All σ,

(4) $$\varepsilon ˙=A{\left[\sin\left(\alpha \sigma \right)\right]}^{n}\exp(-\frac{Q}{RT})$$

where σ is the flow stress, ε˙ is the strain rate, R is the universal gas constant, *T* is the absolute temperature, and *Q* is the activation energy of deformation, $A_{1}{,\;A}_{2},\;A,\;\alpha ,\;\beta ,\;n_{1}$, which are constants that depend on the material’s properties and meet the relationship shown in Equation (5).

(5) $$\alpha =\frac{\beta }{n_{1}}$$

(6) $$Z=\varepsilon ˙\exp\left(\frac{Q}{RT}\right)={A[\sin\left(\alpha \sigma \right)]}^{n}$$

Then, using the definition of the hyperbolic sine function to solve Equation (6), the flow stress σ can be written as a function shown in Equation (7) of the *Z* parameter.

(7) $$\sigma =\frac{1}{\alpha }ln\{{\left(\frac{Z}{A}\right)}^{\frac{1}{n}}+[{{\left(\frac{Z}{A}\right)}^{\frac{2}{n}}+1]}^{\frac{1}{2}}\}$$

The natural logarithm of Equations (2)–(4) was taken respectively, and the obtained expression is shown in Equations (8)–(10).

(8) $$\ln\varepsilon ˙=lnA_{1}+n_{1}ln\sigma -\frac{Q}{RT}$$

(9) $$\ln\varepsilon ˙=lnA_{2}+\beta \sigma -\frac{Q}{RT}$$

(10) $$\ln\varepsilon ˙=nln[\sinh\left(\alpha \sigma \right)]+lnA-\frac{Q}{RT}$$

Equation (10) is deformed to obtain Equation (11).

(11) $$ln\left[\sinh\left(\alpha \sigma \right)\right]=\frac{Q}{nR}\cdot \frac{1}{T}-\frac{\ln\varepsilon ˙-\ln A}{n}$$

The data points of the peak stress shown in Table 2 are substituted into Equations (8)–(11) for fitting Arrhenius model parameters.

**Figure 4:**
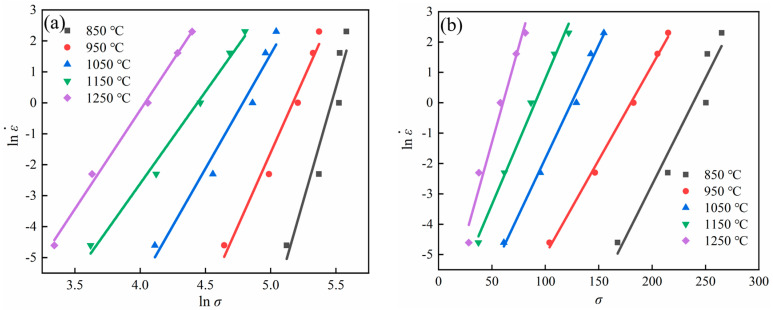

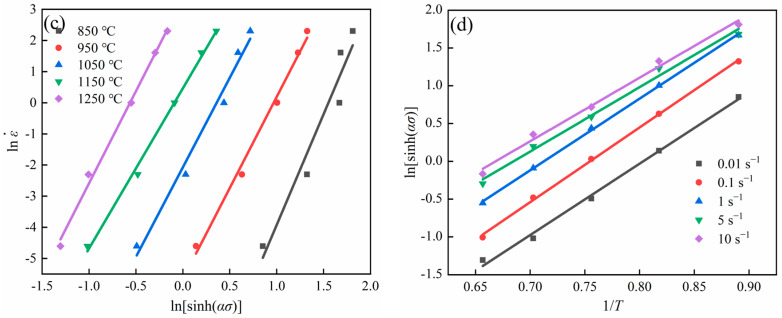
Relationships of (**a**) ln⁡ε˙-lnσ; (**b**) ln⁡ε˙-σ; (**c**) ln⁡ε˙-sinh⁡(ασ); (**d**) $ln\left[\sinh\left(\alpha \sigma \right)\right]-\frac{1}{T}$.

Based on Equations (8) and (9), the relationships of ln⁡ε˙−lnσ and ln⁡ε˙−σ are plotted in Figure 4a,b. Then, the values of the parameter $n_{1}$ and β can be calculated with the slope of lines in Figure 4a,b, and $n_{1}$ = 8.77523, β = 0.083108 can be obtained. Equation (5) can represent the relationship between the stress level parameter and the stress index, so the α = 0.017847 can be obtained.

Similarly, ln⁡ε˙-sinh⁡(ασ) scatterplot can be made with Equation (10), and a linear fit was performed. The results are shown in Figure 4c, giving *n* = 5.957176.

The substitution of the flow stress value under peak conditions into Equation (11) enabled the linear fitting of the scatters of $\ln\left[\sin h\left(\alpha \sigma \right)\right]-\frac{1}{T}$, as shown in Figure 4d. The resulting graph indicated that the average slope of the linear fitting curve was 8.39921, with an intercept average of −5.61362. As a result, the average value of hot deformation activation energy was calculated, being *Q* = 452,648 kJ/mol, and ln A is calculated to be 38.98675.

The α, n, Q, A values calculated using linear fitting were substituted into Equation (4) to obtain the Arrhenius constitutive equation for thermal deformation of the 26CrMo7S steel under peak conditions, as shown in Equation (12).

(12) $$\varepsilon ˙=5.249\times 10^{17}{\left[\sinh\left(0.009471\sigma \right)\right]}^{5.957}exp(-\frac{452648.321}{8.314T})$$

In Equation (1), the calculated hot deformation activation energy *Q* value was substituted to obtain the parameter Equation (13).

(13) $$Z=\varepsilon ˙\exp\left(\frac{452648.321}{8.314T}\right)$$

In Equation (7), substituting the calculated parameters can obtain the stress equation of 26CrMo7S steel expressed in *Z* parameters under peak conditions, as shown in Equation (14).

(14) $$\sigma =\frac{1}{0.009471}ln\{{\left(\frac{Z}{A}\right)}^{\frac{1}{5.957}}+[{{\left(\frac{Z}{A}\right)}^{\frac{2}{5.957}}+1]}^{\frac{1}{2}}\}$$

#### 3.2.2. Accuracy Verification of Constitutive Equation

Error analysis of the model prediction and experimental values can further describe the accuracy of the above constitutive equations. The correlation coefficient *r* can express the linear strength between the predicted and experimental values, and r can be computed as shown in Equation (15).

(15) $$r=\frac{\sum_{i=1}^{N}(\sigma_{exp}^{i}-{\bar{\sigma }}_{exp})(\sigma_{pre}^{i}-{\bar{\sigma }}_{pre})}{\sqrt{\sum_{i=1}^{N}{\left(\sigma_{exp}^{i}-{\bar{\sigma }}_{exp}\right)}^{2}{\left(\sigma_{pre}^{i}-{\bar{\sigma }}_{pre}\right)}^{2}}}$$

The deviation between the predicted and experimental values can be reflected by the absolute error (AE), which can be expressed as shown in (16).

(16) $$AE=|\sigma_{exp}^{i}-\sigma_{pre}^{i}|$$

The relative error (RE) between the predicted value and the experimental value can be calculated with the average relative error (AAER), as is shown in Equation (17).

(17) $$AARE=\frac{1}{N}\sum_{i=1}^{N}\left|\frac{\sigma_{exp}^{i}-{\bar{\sigma }}_{pre}}{\sigma_{exp}^{i}}\right|\times 100\%$$

In the above equation, *N* represents the number of experimental data; $\sigma_{exp}^{i}$ represents the experimental value of flow stress, ${\bar{\sigma }}_{exp}$ represents the average of the experimental value, MPa; $\sigma_{pre}^{i}$ represents the predicted value of rheological stress, and ${\bar{\sigma }}_{pre}$ represents the average of the predicted values, MPa.

**Figure 5:**
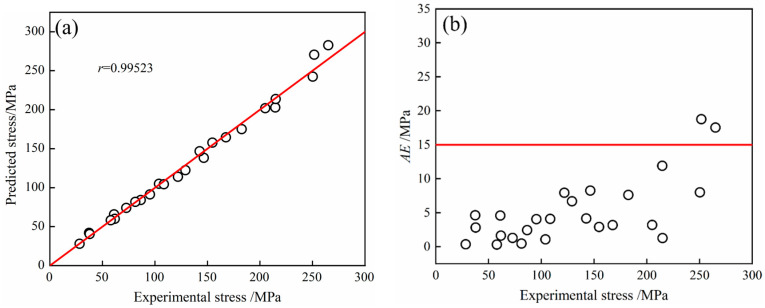
Calculation of the error between the predicted value of the peak stress and the experimental value: (**a**) r; (**b**) *AE*.

The calculated constitutive equation enables the prediction of flow behavior under different deformation conditions. The peak stress prediction values were calculated with Equation (14), and the experimental data were substituted into Equations (15)–(17). The corresponding the correlation coefficient and absolute error were obtained, as illustrated in Figure 5, by taking the peak stress data under 36 deformation conditions as samples and comparing the experimental and predicted values. Figure 5a displays the correlation coefficient between the experimental and predicted values, r = 0.99523. Figure 5b shows the absolute error distribution, indicating that the peak stress value calculated with the equation had little difference from the experimental value. Overall, the peak stresses predicted with the constitutive equation model closely matched the experimental values.

It can be seen from Table 3 that the points with a relative error of less than 10% accounted for 91.7%. In terms of the value in Table 3, it can be calculated that the average relative error of all data was 4.4%, which indicated that the Arrhenius constitutive model had a high accuracy in predicting the peak stress of the 26CrMo7S steel.

### 3.3. Processing Map

#### 3.3.1. Dynamic Material Model Theory

**Figure 6:**
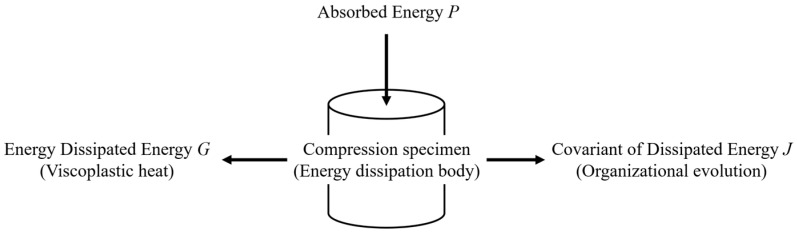
The illustration of the energy dissipation mechanism.

Based on the Dynamic Material Model (DMM) theory, the material dissipates as a power dissipator during hot deformation. The absorbed energy is mainly dissipated in two forms: one is the deformation viscoplastic heat generated at high temperature and high strain rate, which is dissipated through heat; the other one is the microstructure evolution, which is dissipated through dynamic recovery, dynamic recrystallization, hole formation, and phase transformation. Figure 6 illustrates the energy dissipation mechanism.

As shown in Figure 6, P is the total power absorbed by the compression specimen during the hot deformation process and can be divided into two parts: G and J. G is the power consumption of the deformation viscoplastic heat; J is the power consumption of the microstructure evolution. The relationship among them can be expressed with Equation (18), including flow stress (*σ*), strain (*ε*), and strain rate ($\dot{\varepsilon }$).

(18) $$P=\sigma \dot{\varepsilon }=J+G=\int_{0}^{\sigma }\dot{\varepsilon }d\sigma +\int_{0}^{\dot{\varepsilon }}\sigma d\dot{\varepsilon }$$

According to the Prasad criterion, the material’s flow stress and strain rate have an exponential function for specific strain and deformation temperature, which can be expressed with Equation (19).

(19) $$\sigma =K{\varepsilon ˙}^{m}$$

K is the material constant, and *m* is the strain rate sensitivity coefficient. Also, they are decisive for the allocation of P in the two parts, G and J.

Deriving from $\dot{\varepsilon }=\frac{\partial J}{\partial \sigma },\;\;\sigma =\frac{\partial G}{\partial \dot{\varepsilon }}$ and Equations (19) and (20) can be obtained.

(20) $$m=\frac{\partial J}{\partial G}=\frac{\dot{\varepsilon }d\sigma }{\sigma d\dot{\varepsilon }}=\frac{\partial (ln\sigma )}{\partial G(ln\dot{\varepsilon })}=\frac{\partial (lg\sigma )}{\partial (lg\dot{\varepsilon })}$$

**Figure 7:**
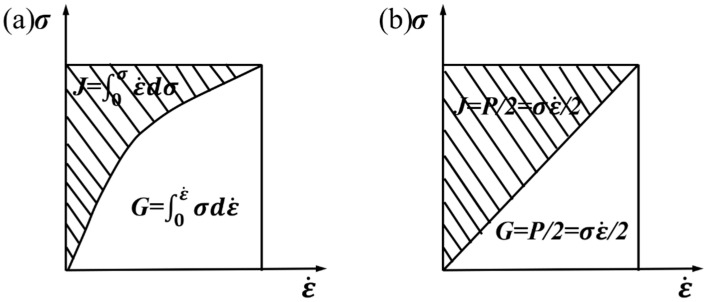
Material system energy dissipation map. (**a**) Nonlinear dissipation (0 < *m* < 1); (**b**)linear dissipation (*m* = 1).

According to the dynamic material models, when m ≤ 1, the material does not exhibit energy dissipation behavior. When the m-value is between 0 and 1, it indicates that a nonlinear dissipation process occurs, as shown in Figure 7a. When *m* = 1, the material exhibits the ideal linear dissipation process, as shown in Figure 7b. At this point, the material dissipation covariant J reaches the maximum value, which is equivalent to the viscoplastic heat G due to plastic deformation, exhibiting a Newtonian viscous fluid state. 

Prasad et al. [32,33,34] introduced the power dissipation factor *η* to represent the microscopic deformation mechanism in the hot deformation process. The *η* value is the ratio between the J and *J*_max_, as shown in Equation (21).

(21) $$\eta =\frac{J}{J_{max}}$$

where *η* is the power dissipation factor, *J* is the dissipative covariate in the nonlinear dissipation process, and *J_max_* is the ideal linear dissipative covariate.

(1) Prasad et al. [34] proposed that the relationship between σ and $\dot{\varepsilon }$ was a power exponent, and the dynamic constitutive equation could be expressed as $\sigma =K{\varepsilon ˙}^{m}$, so *J* could be expressed as Equation (22).

$$J=\int_{0}^{\sigma }\dot{\varepsilon }d\sigma =\int_{0}^{\dot{\varepsilon }}\dot{\varepsilon }d(K{\dot{\varepsilon }}^{m})=\int_{0}^{\dot{\varepsilon }}mK{\dot{\varepsilon }}^{m}d\dot{\varepsilon }=K\frac{m}{m+1}{\left.{\dot{\varepsilon }}^{m+1}\right|}_{0}^{\dot{\varepsilon }}$$

(22) $$=\frac{m}{m+1}K{\dot{\varepsilon }}^{m+1}=\frac{m}{m+1}\sigma \dot{\varepsilon }$$

Thus, according to Equation (22), when *m* = 0, J = 0; when *m* = 1, J = $J_{max}$ = $\frac{1}{2}\sigma \dot{\varepsilon }$. The power dissipation factor *η*, the dimensionless parameter, can be expressed with Equation (23).

(23) $$\eta =\frac{J}{J_{max}}=\frac{\frac{m}{m+1}\sigma \dot{\varepsilon }}{\frac{1}{2}\sigma \dot{\varepsilon }}=\frac{2m}{m+1}$$

The flow instability criterion is shown in Equation (24).

(24) $$\xi \left(\dot{\varepsilon }\right)=\frac{\partial ln(\frac{m}{m+1})}{\partial \dot{\varepsilon }}+m<0$$

where $\xi \left(\dot{\varepsilon }\right)$ represents the instability coefficient. When $\xi \left(\dot{\varepsilon }\right)$ is negative, it indicates that the material is undergoing plastic deformation in an unstable state, where instability may occur. The physical interpretation is that the rate of entropy produced by the system matches the need for additional entropy. If the strain rate applied to the system exceeds the system’s entropy rate, an unstable flow may occur in the local area.

(2) The Murty criterion believed that only $\dot{\varepsilon }<{\dot{\varepsilon }}_{min}$, σ and $\dot{\varepsilon }$ is a power exponential relationship [35]. Then, G can be expressed as Equation (25).

$$G=\int_{0}^{\dot{\varepsilon }}\sigma d\dot{\varepsilon }=\int_{0}^{{\dot{\varepsilon }}_{min}}K{\dot{\varepsilon }}^{m}d\dot{\varepsilon }+\int_{{\dot{\varepsilon }}_{min}}^{\dot{\varepsilon }}\sigma d\dot{\varepsilon }=K\frac{1}{m+1}{\left.{\dot{\varepsilon }}^{m+1}\right|}_{0}^{{\dot{\varepsilon }}_{min}}+\int_{{\dot{\varepsilon }}_{min}}^{\dot{\varepsilon }}\sigma d\dot{\varepsilon }$$

(25) $$={\left.\frac{\sigma \dot{\varepsilon }}{m+1}\right|}_{\dot{\varepsilon }={\dot{\varepsilon }}_{min}}+\int_{{\dot{\varepsilon }}_{min}}^{\dot{\varepsilon }}\sigma d\dot{\varepsilon }$$

When $\dot{\varepsilon }>{\dot{\varepsilon }}_{min}$, the power dissipation factor can be integrated directly by utilizing the second term of Equation (25) to establish a corresponding three-spline interpolation function or a constitutive model equation. The expression for the power dissipation factor is shown in Equation (26).

(26) $$\eta =\frac{J}{J_{max}}=\frac{P-G}{P/2}=2\left(1-\frac{G}{P}\right)=2\left[1-\frac{1}{\sigma \dot{\varepsilon }}({\left.\frac{\sigma \dot{\varepsilon }}{m+1}\right|}_{\dot{\varepsilon }={\dot{\varepsilon }}_{min}}+\int_{{\dot{\varepsilon }}_{min}}^{\dot{\varepsilon }}\sigma d\dot{\varepsilon })\right]\;\;$$

The corresponding flow instability criterion is shown in Equation (27).

(27) $$\xi \left(\dot{\varepsilon }\right)=2m-\eta <0$$

Various criteria, such as the Malas and Gegel stabilization zone and Semiatin instability criteria, are useful in predicting the behavior of specific materials [36,37]. However, the Murty criterion is particularly versatile as it can be applied to any flow stress–strain curve and is notable for its simplicity, speed, and thorough analysis. Therefore, the Murty instability criterion is utilized to establish the hot processing feasible domain of the 26CrMo7S steel.

#### 3.3.2. The Hot Processing Maps of 26CrMo7S Steel

**Figure 8:**
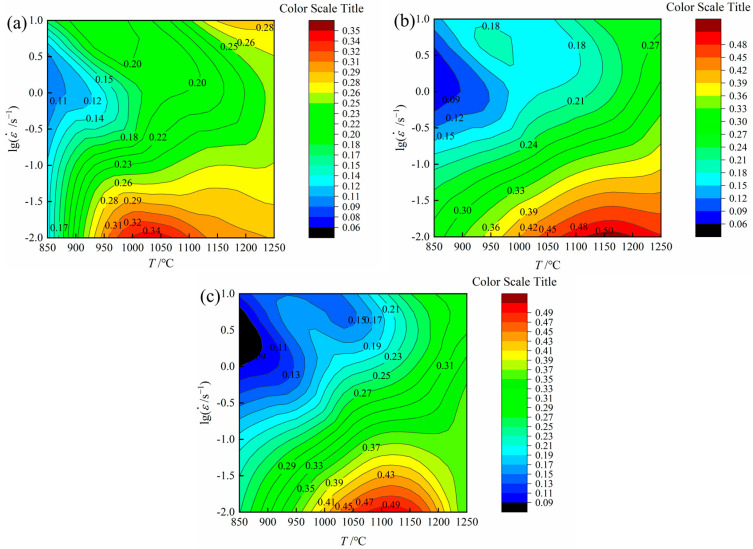
Energy dissipation maps with strain of (**a**) 0.1; (**b**) 0.4; (**c**) 0.7.

The hot processing maps can be obtained by combining the power dissipation and flow instability maps. The contour lines are used to represent different power dissipation factor *η* values. The flow instability values are computed through the flow instability criterion. The hot processing safety zone region is the area with high power dissipation. For example, when the strain was 0.1 (Figure 8a), *η* peaked at a deformation temperature of 1010~1050 °C and a strain rate of 0.01~10^−1.969^ s^−1^, approximately 0.34. For a strain of 0.4 (Figure 8b), *η* peaked at a deformation temperature of 1120~1200 °C and a strain rate of 0.01~10^−1.916^ s^−1^, approximately 0.50. *η* also climbed to its maximum when the strain was 0.7 (Figure 8c), at a deformation temperature of 1074~1152 °C and a strain rate of 0.01~10^−1.909^ s^−1^, approximately 0.49. The power dissipation factor exhibited relative stability during the strain change, suggesting that the 26CrMo7S steel entered a stable deformation stage at a strain of 0.4, with flow stress also remaining stable. These results show that the higher the value of *η*, the more beneficial the material’s machinability, and the deformation domain could be in optimal condition for the hot deformation process [38,39,40,41].

**Figure 9:**
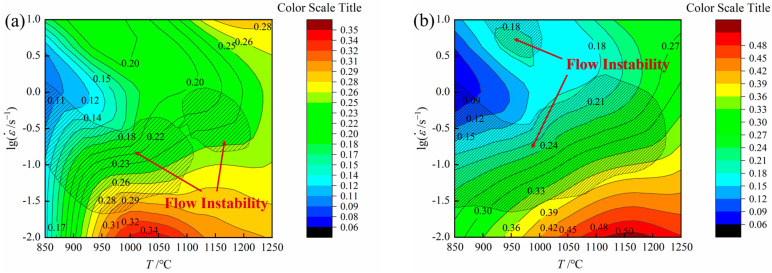

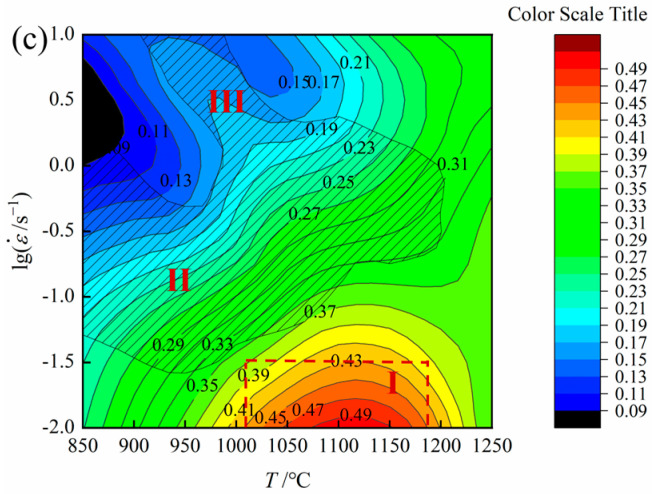
Hot processing maps of 26CrMo7S steel with strains of (**a**) 0.1; (**b**) 0.4; (**c**) 0.7.

Figure 9 presents the hot processing maps of 26CrMo7S steel at different strains. Notably, the *η* value increased successively as the temperature rose and the strain rate decreased because the energy consumption elevates during deformation with increasing degrees of dynamic recovery and recrystallization, which makes the experimental steel have few internal defects and fine grain refinement. The hot processing maps at different strains displayed comparable features that surfaced in the details. As shown in Figure 9a, insufficient dynamic behavior was observed at low strain due to fewer degrees of deformation, and large power dissipation was not generated. The areas of plastic flow instability mainly occurred at a deformation temperature range of 850~1110 °C with a strain rate of 10^−1.72^~10^0.2^ s^−1^ and at a temperature range of 1100~1215 °C with a strain rate of 10^−0.75^~10^0.1^.

With increasing strain, the region where plastic instability occurred expanded. For example, a strain of 0.4 resulted in plastic instability in regions where the deformation temperature ranged from 850 to 1223 °C and the strain rate from 10^−1.66^ to 10^0.221^ s^−1^. Similarly, the high strain zone with a temperature range of 900~1005 °C and a strain rate between 10^0.466^ and 10 s^−1^ also showed characters of local instability (Figure 9b). These areas suggested that high strain rates could easily lead to localized instability in the material.

The high-power dissipation occurred mostly in areas with high temperatures and low strain rates because it is more beneficial for dislocation to migrate at such temperatures. A strain of 0.7 resulted in peak *η* values mostly concentrated at a strain rate range of 10^−1.25^~10 s^−1^. Based on these findings, we could identify three typical processing areas (Zone I, II, and III), as shown in Figure 9c. Zone I is the safe processing zone, while Zones II and III represent the plastic flow instability zones. The corresponding deformation temperature, strain rate, and power dissipation factor are shown in Table 4.

### 3.4. Microstructural Observation

Different regions in the processing maps indicated that some specific microstructural evolutions occurred in the samples treated by corresponding processing parameters. To better understand the microscopic evolution characteristics of the samples, various areas from the hot processing maps of strain 0.7 were selected for EBSD testing. 

**Figure 10:**
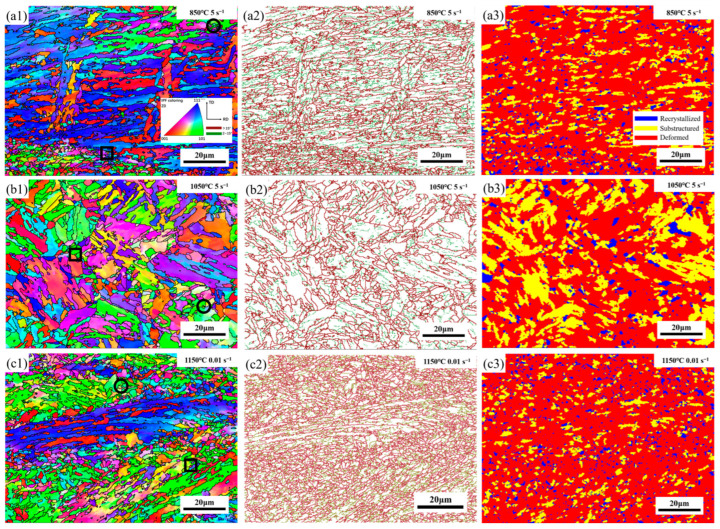
The EBSD images of the specimens under different hot conditions: (**a**) 850 °C, 5 s^−1^; (**b**) 1050 °C, 5 s^−1^; (**c**) 1150 °C, 0.01 s^−1^; (1) IPF map; (2) grain boundary misorientation; (3) recrystallized distribution.

The EBSD results of experimental steel under different thermal conditions are shown in Figure 10. Figure 10(a1–c1) shows the maps of IPF at 850, 1050, and 1150 °C, the grain boundary misorientation Figure 10(a2–c2), and recrystallized distribution maps Figure 10(a3–c3). The different colors in the IPF map stand for different orientations. Additionally, in the maps of grain boundary misorientation, the red and green lines indicate the high-angle grain boundary (HAGB, >15°) and low-angle grain boundary (2° < LAGB < 15°), respectively. It can be seen that LAGBs occur in the center of the origin grain [42]. The maps of recrystallized distribution show the distribution of recrystallized structure, substructure, and deformed structure (Figure 10(a3–c3)). These three structures are represented by the colors blue, yellow, and red, respectively.

Figure 10a shows the microstructure of 26CrMo7S steel deformed at the condition of the temperature of 850 °C and strain rate of 5 s^−1^, which was dissipation low-value region *η* = 0.09. It can be obviously seen that there were recrystallization and grain growth phenomena in Figure 10(a1,a3), which indicated that some grains reached dynamic recrystallization nuclear energy to begin nucleating and growing [43].

Figure 10b shows the deformation microstructure of the sample at a deformation temperature of 1050 °C and a strain rate of 5 s^−1^, which belonged to the flow instability region of medium temperature and high strain rate, and *η* = 0.15. It can be seen that the original grain of the material was significantly elongated, and the dynamic recrystallization (DRX) grew at the original grain boundary. This phenomenon can lead to uneven structure, making mechanical instability during hot deformation more likely. 

When the temperature reached 1150 °C, and the strain rate was 0.01 s^−1^, the power dissipation coefficient *η* hit 0.49; the corresponding EBSD images are shown in Figure 10c. More fine grains occurred, with only a few originally deformed grains remaining, as shown in Figure 10(c1,c3). The original grains were encircled by fine grains, which included a combination of dynamically recrystallized grains and original grains with deformed shapes. 

Based on the recrystallization distribution maps and IPF maps, it can be observed that new dynamically recrystallized grains occur mainly at triple junctions and original boundaries marked respectively in black boxes and circles, as shown in Figure 10(a1–c1) [43]. According to YANG et al. [44], this suggests that the occurrence of DRX is more dominant at these locations. Within these locations, some grains exhibit characteristics of continuous dynamic recrystallization (CDRX), with a color difference from the original deformed grains, as indicated by black boxes in Figure 10(a1–c1). This color difference implies that the grains rotate during the hot deformation process, resulting in varied misorientation. During the process of gradual rotation of grains, new DRX grains are formed due to the continuous absorption of dislocations in LAGBs [45]. In addition, some new DRX grains are formed due to the bulging of grain boundaries with high angles, which can be seen as marked by black circles in Figure 10(a1–c1). This indicates that the deformation mechanism is discontinuous dynamic recrystallization (DDRX) [42,46,47]. 

**Figure 11:**
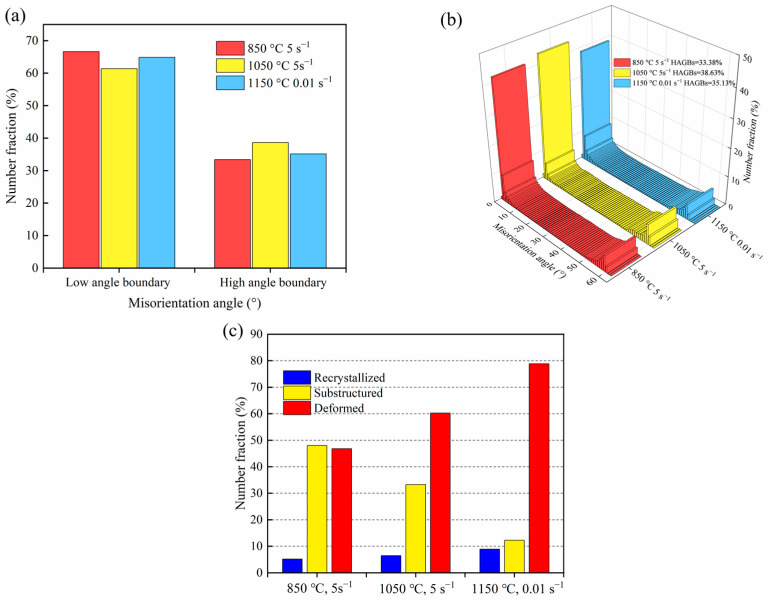
The EBSD results of the specimens: (**a**) statistics of grain boundaries; (**b**) grain boundary distribution; (**c**) recrystallized fraction.

Figure 11 displays the EBSD results of the experimental steel under different thermal conditions. It includes statistics of high- and low-angle grain boundaries (a), grain boundary distribution (b), and recrystallized fraction (c). As shown in Figure 11a,b, the proportion of LAGB is prominent in the grain distribution, while the percentage of HAGB increases with the temperature rise. Specifically, the percentage of HAGB is 33.38%, 38.63%, and 35.13%, respectively, for different thermal conditions. Figure 11c calculates the statistics of the three structures in the recrystallized distribution maps. The proportion of recrystallized structure increases with the temperature increase. The primary microstructure after deformation at 850 °C, 1050 °C, and 1150 °C constituted the substructure with a number fraction of 48%, 33%, and 12%, respectively. The substructure may be related to dislocation cells formed by dislocation stacking [48].

Ultimately, these findings above emphasized that hot processing maps of the 26CrMo7S steel were crucial in forecasting the microstructure of hot deformation.

## 4. Conclusions

The hot deformation behaviors and microstructure of 26CrMo7S steel were investigated in order to understand the hot deformation mechanism and hot processing parameters. The major conclusions are as follows:

(1) Experimental steel has negative temperature sensitivity and positive strain rate sensitivity, and the peak stress decreases with increasing temperature or decreasing strain rate, which is the result of the interaction of dynamic softening and work hardening.
(2) The developed Arrhenius constitutive model has high accuracy in predicting the peak stress of the 26CrMo7S steel. The hot deformation activation energy is 452.648 kJ/mol. The predicted value is strongly linearly correlated with the experimental value, and the correlation coefficient reaches 0.99523.
(3) The suitable domain of hot processing found from the hot processing maps is at a temperature of approximately 1010~1190 °C and a strain rate of 0.01~10^−1.5^ s^−1^, where the peak efficiency *η* exceeds 0.4, and more fine grains occurred, leaving only a few initially deformed grains.

## Figures and Tables

**Table 1:** Chemical composition of the 26CrMo7S steel (wt.%).

C	Si	Mn	Cr	Mo	S	Nb	V	Ti	Fe
0.29	0.20	0.44	0.46	0.80	0.002	0.03	0.035	0.01	Bal.

**Table 2:** The peak stress of the 26CrMo7S steel.

Temperature (°C)	Strain Rate (s^−1^)				
	0.01	0.1	1	5	10
850	167.62	214.65	250.34	251.66	265.05
950	103.87	146.45	182.54	205.08	214.96
1050	61.093	95.263	129.03	142.55	154.77
1150	37.35	61.73	86.57	108.49	121.86
1250	28.28	37.78	57.91	72.72	81.25

**Table 3:** Relative error between peak stress prediction and experimental value/%.

Temperature (°C)	Strain Rate (s^−1^)				
	0.01	0.1	1	5	10
850	4.440662	6.909438	1.643345	7.233389	5.519126
950	3.222338	7.286127	2.912853	1.495324	1.163817
1050	8.713637	7.552891	5.727646	1.910679	1.577422
1150	11.75282	5.947154	5.711019	6.128593	8.564525
1250	7.821027	6.554237	2.812459	2.511807	1.844026

**Table 4:** The range of temperature, strain rate, and power dissipation efficiency of Zones I–III.

Zone	Temperature (°C)	Strain Rate (s^−1^)	*η*
I	1010~1190	0.01~10^−1.5^	0.43~0.49
II	850~1203	10^−1.57^~10^0.38^	0.09~0.33
III	905~1056	10^0.34^~10	0.15~0.17

## Data Availability

Research data are not shared.
